# The role of automated insulin delivery technology in diabetes

**DOI:** 10.1007/s00125-024-06165-w

**Published:** 2024-05-13

**Authors:** Charlotte K. Boughton, Roman Hovorka

**Affiliations:** Wellcome-Medical Research Council Institute of Metabolic Science, University of Cambridge, Cambridge, UK

**Keywords:** Artificial pancreas, Automated insulin delivery, Closed-loop, Diabetes technology, Review, Type 1 diabetes, Type 2 diabetes

## Abstract

The role of automated insulin delivery systems in diabetes is expanding. Hybrid closed-loop systems are being used in routine clinical practice for treating people with type 1 diabetes. Encouragingly, real-world data reflects the performance and usability observed in clinical trials. We review the commercially available hybrid closed-loop systems, their distinctive features and the associated real-world data. We also consider emerging indications for closed-loop systems, including the treatment of type 2 diabetes where variability of day-to-day insulin requirements is high, and other challenging applications for this technology. We discuss issues around access and implementation of closed-loop technology, and consider the limitations of present closed-loop systems, as well as innovative approaches that are being evaluated to improve their performance.

**Graphical Abstract:**

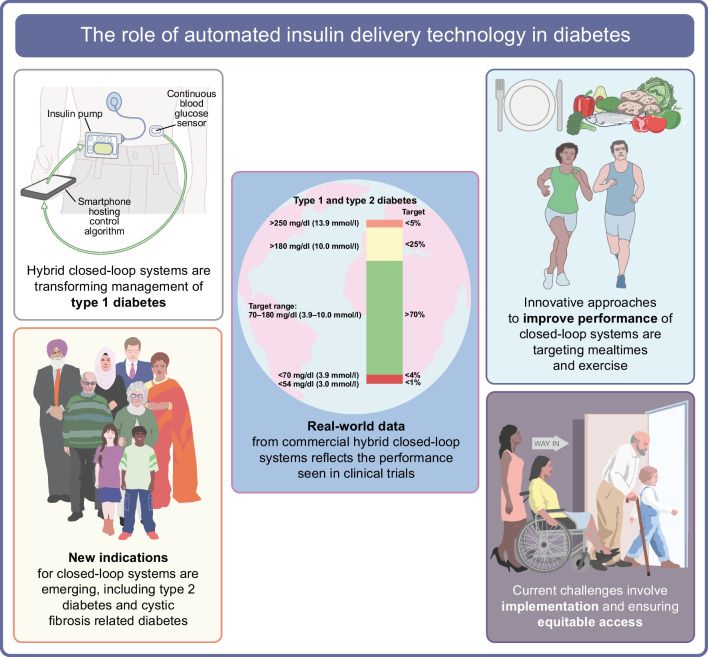

**Supplementary Information:**

The online version contains a slideset of the figures for download, which is available to authorised users at 10.1007/s00125-024-06165-w.

## Introduction

Automated insulin delivery systems, also called closed-loop systems or artificial pancreases, have successfully transitioned from the research bench to becoming the standard of care for people with type 1 diabetes. This success story is at least in part due to the need for improved treatment strategies and reduced disease burden for the growing population living with diabetes.

Despite the increasing use of insulin pumps and glucose sensors by people with type 1 diabetes, the introduction of new oral and non-insulin injectable therapies for type 2 diabetes, and the continual advancements in modern insulin analogues, registry data from the USA and Europe estimate that the ADA recommended glycaemic target (HbA_1c_ <53 mmol/mol [7%] without significant hypoglycaemia) is met by only a quarter of people with type 1 diabetes and half of those with type 2 diabetes [1, 2]. This highlights a clear need to improve diabetes management approaches and outcomes for both type 1 and type 2 diabetes to prevent the risk of micro- and macrovascular complications and hospital admissions. However, resources, including access to healthcare professionals, are limited and the demand is growing. Globally, 1 in 10 adults are living with diabetes (537 million) and this number is predicted to rise by 100 million over the next 10 years [3].

**Fig. 1:**
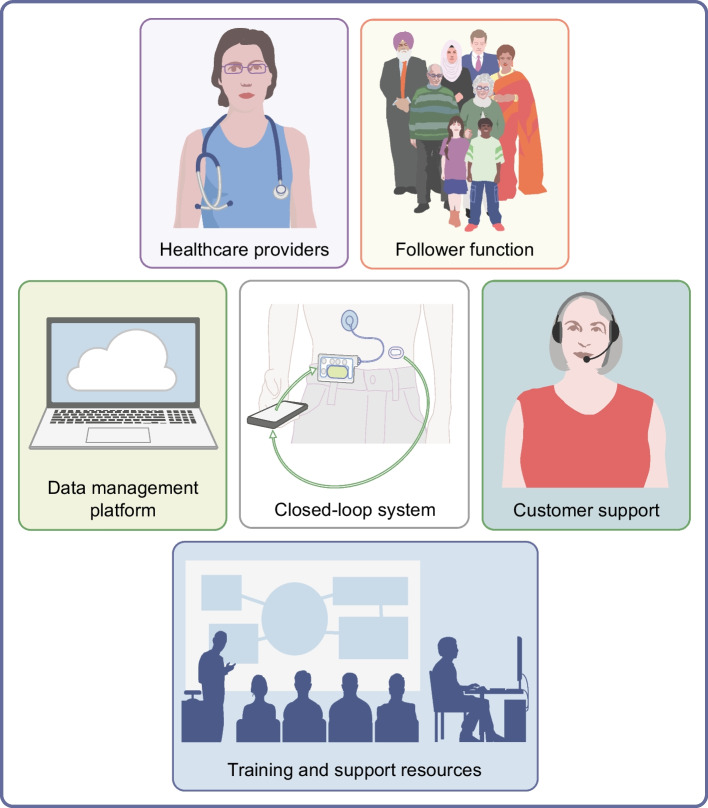
The closed-loop ecosystem. Before starting to use a closed-loop system users require training and support on the devices. Once users are on board, data from the closed-loop system is uploaded to a data management platform and can be visualised in real-time by caregivers/family members (follower function) and by healthcare providers to support optimisation of diabetes management. In the event of device issues, users will need to contact customer support for assistance. This figure is available as part of a downloadable slideset

Closed-loop systems are addressing this need for improved diabetes management, and a related infrastructure required to effectively deliver these solutions, diabetes technology ‘ecosystems’, has emerged. The ecosystems comprise not only the hardware components of the system (i.e. insulin pump and glucose sensor) and the algorithm, but also training resources for users and healthcare professionals, customer support, data-sharing platforms and follower functionality allowing relatives or caregivers to directly view data on their own device, which all impact on the user experience (Fig. 1).

## Clinical benefits of closed-loop systems for people living with diabetes

The benefits of closed-loop systems on glucose management are well established across people of all ages with type 1 diabetes, including pregnant women [4]. Hybrid closed-loop systems, requiring meal announcement, where the user needs to calculate and tell the system their carbohydrate intake, while insulin delivery between meals and overnight is automated, improve glucose management without increasing time spent in hypoglycaemia, or in some cases reduce hypoglycaemia. Meta-analyses undertaken from trial data in adults and in children show that hybrid closed-loop systems outperform non-automated systems with improvements in time spent in target glucose range of approximately 8–12 percentage points, reduced time spent in hyperglycaemia, reduced mean glucose and either a reduction or no increase in time in hypoglycaemia [5–7]. User characteristics that influence the clinical impact of hybrid closed-loop technology include baseline glucose management, with the greatest improvements observed where the baseline HbA_1c_ is highest and time in range lowest [8, 9]. Optimal performance with hybrid closed-loop systems is associated with increased bolus frequency and a higher proportion of total daily insulin delivered as a bolus [10, 11].


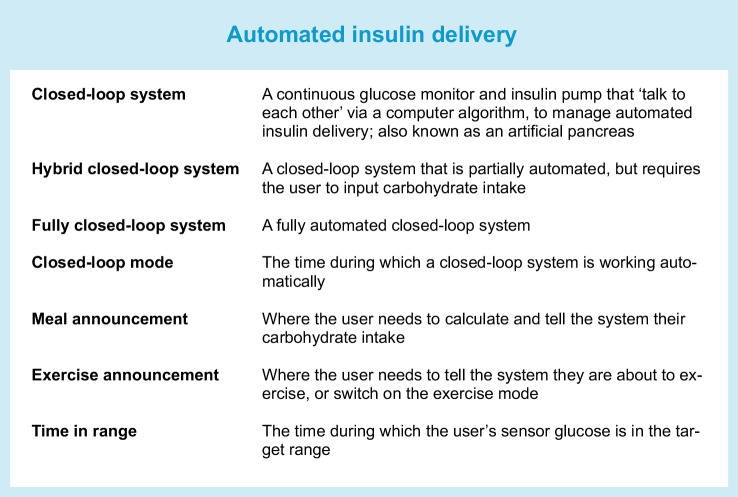


The evidence evaluating closed-loop systems for people with type 2 diabetes is less well established, but interest in this area is growing. Small randomised controlled crossover trials have shown improved glucose management without an increase in hypoglycaemia, compared with standard insulin therapy, in adults with type 2 diabetes and sub-optimal glucose management at baseline using a fully automated closed-loop system (CamAPS HX, CamDiab, UK), which does not require any meal announcement, in an outpatient setting [12, 13]. The same fully automated closed-loop system has also shown safety and efficacy in inpatients during acute hospital admissions [14, 15]. The benefit of a fully closed-loop system in this setting is that it removes the need for healthcare professional input for insulin dosing decisions, which are often inaccurate due to the unpredictability of these situations. Hybrid closed-loop systems (Control-IQ, Tandem, CA, USA and Omnipod 5, Insulet, MA, USA) have also shown feasibility in adults with type 2 diabetes in an outpatient setting in non-randomised studies and real-world data [16, 17]. More severe insulin resistance with greater daily insulin requirements in those with type 2 diabetes may necessitate insulin pumps with larger reservoir capacity compared with those currently being used for people with type 1 diabetes, or use of concentrated insulins in the closed-loop system.

Automated insulin delivery has also been evaluated in a small study of people with cystic fibrosis related diabetes (CFRD) over a period of 14 days, where there was improved time in target glucose range without an increase in time spent in hypoglycaemia compared with usual care [18].

## Psychosocial impact of closed-loop systems for people living with diabetes

Equally, or perhaps more importantly than the clinical benefits of closed-loop systems, are the potential psychosocial benefits in reducing the burden of diabetes management. Improvements in person-reported outcome measures associated with closed-loop technology have been inconsistent, which may relate to study design, population and length of follow-up, but all studies have shown that, even if there was no improvement in quality-of-life, there was no deterioration [19]. Specific benefits reported by closed-loop users include reduced diabetes distress, reduced fear of hypoglycaemia and improved quality of sleep. Meta-analysis data of person-reported outcome measures from hybrid closed-loop trials longer than 3 months found decreased diabetes distress and a tendency for reduced fear of hypoglycaemia but no significant difference in treatment satisfaction [20]. Perceived drawbacks include connectivity problems, automation-related errors, pump glitches and other issues associated with insulin pumps. Evaluation of fully automated closed-loop systems may result in further improvements in quality-of-life and treatment satisfaction.

Qualitative interviews provide richer and more in-depth information on user experiences of closed-loop systems. Many users, including children and adults with type 1 diabetes, describe the technology as ‘life-changing’, associating it with improved well-being and facilitating a greater sense of normality [21–23]. For some people it has even facilitated a return to paid employment. The psychosocial benefits of closed-loop systems have also been reported to extend to family members including parents, partners and children of people with diabetes [21, 24]. Pregnant women using closed-loop systems described how it lessened the demands of diabetes management, enabling them to feel more normal and sleep better [25]. All women reported more enjoyable pregnancy experiences as a result of using closed-loop technology.

It is important to note that reports of user experiences from closed-loop studies are limited by the relative lack of ethnic and socioeconomic diversity of the research study populations [26]. There are also limited reports on the psychosocial impact of closed-loop systems from real-world settings, and very limited reports on the psychosocial impact of closed-loop technology in adults with type 2 diabetes or those with CFRD [12, 18].

## Commercially available closed-loop systems for people with type 1 diabetes

The current commercially available closed-loop systems are hybrid systems requiring the user to announce meals. Each system has distinct features that may benefit specific user groups (Table 1).

**Table 1:** Features of commercially available hybrid closed-loop systems

	MiniMed 670G/770G/780G (Medtronic)	Control-IQ (Tandem)	DBLG1 (Diabeloop)	CamAPS FX (CamDiab)	Omnipod 5 (Insulet)	iLet bionic pancreas (BetaBionics)
Insulin pump	MiniMed 670G/770G/780G	t:slim X2	Kaleido pump	YpsoPump Dana pump	Omnipod patch pump	iLet pump
Glucose sensor	Guardian 3 (requires calibration)/ Guardian 4	Dexcom G6/G7 Freestyle Libre 2 Plus	Dexcom G6	Dexcom G6 Freestyle Libre 3	Dexcom G6 FreeStyle Libre 2 Plus	Dexcom G6/G7
Algorithm	PID with insulin feedback. 780G also contains automated bolus insulin corrections	MPC algorithm uses pre-programmed basal rates and correction factors with automated bolus insulin corrections	MPC inspired, based on machine-learning within a physiological framework with an expert system and self-learning algorithms	MPC adaptive algorithm calculates and adjusts insulin sensitivity, carbohydrate bioavailability and active insulin time	MPC algorithm calculates adaptive basal insulin rate based on recent total daily insulin dose	Dosing decision software made up of three algorithms (basal insulin, bolus correction & meal aware algorithm)
Algorithm setup	TDD, weight, ICR, CF, basal rate	TDD, weight, ICR, CF, basal rate	TDD, weight, typical meal carbohydrate content, basal rate	TDD, weight	TDD, ICR, CF, basal rate	Weight
Adaptive learning	✓ Overall	X	✓	✓ Overall, diurnal, meals	✓ Basal rate for each Pod	✓ Overall, meal announcement algorithm
Bolusing from phone	X	X	X	✓	X	X
Personal glucose target	780G: 5.5, 6.1, 6.7 mmol/l	Sleep mode: 6.1–6.7 mmol/l	5.6–7.2 mmol/l	4.4–11.0 mmol/l	6.1–8.3 mmol/l	6.1, 6.7, 7.2 mmol/l
Activity/ease-off mode	✓	✓	✓	✓	✓	X
Boost mode	X	X	✓ (aggressiveness)	✓	X	X
Remote monitoring	780G: ✓ CareLink Connect app	✓ CGM only Dexcom Follow app	X	✓ CamAPS Companion app	✓ CGM only Dexcom Follow app Omnipod VIEW app	✓ CGM only Dexcom Follow app
Automated data upload	780G: ✓ CareLink	X	✓ YourLoops	✓ Glooko	✓ Glooko	✓ Beta Bionics Cloud
Insulin	Rapid	Rapid	Rapid	Rapid & ultra-rapid	Rapid	Rapid & Fiasp
Licence	T1D age 7+ (FDA and CE mark)	Diabetes requiring insulin age 6+ (FDA and CE mark)	T1D age 18+ (CE mark)	Diabetes requiring insulin age 1+ including pregnancy (CE mark)	T1D age 2+ (FDA and CE mark)	T1D age 6+ (FDA)

CE, Conformité Européenne; CF, correction factor; ICR, insulin-to-carbohydrate ratio; MPC, model predictive control; PID, proportional integral derivative; T1D, type 1 diabetes; TDD, total daily insulin dose

The first-generation Medtronic hybrid closed-loop system (MiniMed 670G, Medtronic, CA, USA) has been superseded by the second generation MiniMed 780G, which has improved usability (fewer auto mode exits and alarms) and improved glucose management with automated bolus insulin corrections and a meal detection module in addition to the existing features of the MiniMed 670G [27]. The MiniMed 780G also benefits from the calibration-free Guardian 4 sensor and compatibility with Apple Watch. Its licence is for people with type 1 diabetes aged 7 years and above.

The Control-IQ hybrid closed-loop algorithm, hosted on the t:slim X2 insulin pump (Tandem, CA, USA), uses pre-programmed basal rates, correction factors and carbohydrate-to-insulin ratios as part of its algorithm inputs, while the correction target and active insulin time are fixed in the system [28]. The system benefits from a choice of compatible sensors (Dexcom G6/G7, Dexcom, CA, USA; Freestyle Libre 2 Plus, Abbott Diabetes Care, CA, USA). Its licence is for people with diabetes requiring insulin aged 6 years and above.

The Diabeloop DBLG1 hybrid closed-loop algorithm (Diabeloop, France), hosted on a dedicated controller handset, is based on machine-learning within a physiological framework with an expert system and self-learning algorithm. DBLG1 allows users to customise the algorithm through ten different settings including target glucose level and hypoglycaemia threshold [29]. Its licence is for people with type 1 diabetes aged 18 years and above. Diabeloop for highly unstable type 1 diabetes (DBLHU) is a hybrid closed-loop system derived from the DBLG1, which is licenced for the indication of unstable diabetes.

CamAPS FX (CamDiab, UK) is an Android app-based hybrid closed-loop system designed to be interoperable with different insulin pumps (YpsoPump, Ypsomed, Switzerland; Dana, Sooil, South Korea) and sensors (Dexcom G6 and Freestyle Libre 3). The system requires weight and total daily insulin dose for initialisation, while the adaptive algorithm calculates and adjusts insulin sensitivity, carbohydrate bioavailability and active insulin time. The system benefits from a wide range of target glucose settings (4.4 to 11.1 mmol/l) and can be used with rapid and ultra-rapid insulins. Its licence is for people with diabetes aged 1 year and upwards and includes pregnant women.

Omnipod 5 is the first hybrid closed-loop system that utilises a tubeless/patch pump receiving data directly from the Dexcom G6 glucose sensor. The adaptive features of the control algorithm, hosted on a dedicated controller handset, use the recent total daily insulin dose to update the adaptive basal rate, giving a new baseline for automated insulin delivery with each pump change. Target glucose is the only adjustable setting (6.1 to 8.3 mmol/l) that directly impacts automated insulin delivery. Its licence is for people with type 1 diabetes aged 2 years and above.

The iLet bionic pancreas (BetaBionics, CA, USA) uses an adaptive closed-loop algorithm that initialises with the user’s body weight and requires no additional insulin dosing parameters [30]. This eliminates the need to determine pre-set basal rates, insulin-to-carbohydrate ratios or correction factors, which are all calculated by the algorithm. The system simplifies mealtimes with a meal announcement feature allowing users to estimate the amount of carbohydrates in the meal. Its licence is for people with type 1 diabetes aged 6 years and older.

The lengthy and complex development and approval processes required for commercial closed-loop systems, along with access and reimbursement limitations, gave rise to open-source closed-loop systems [31]. These so-called ‘do-it-yourself’ systems have been created and supported by online communities who generate resources providing detailed instructions for setup and use. Open-source systems are designed for considerable user customisation, allowing adjustment of algorithm parameters, include specific functionalities and have a range of compatible devices and user interfaces. Limitations include the need for a level of health and digital literacy above what is usual in the general population to set up and maintain the system, with updates having to be performed manually by the user.

## Real-world evidence for closed-loop systems in type 1 diabetes

Real-world data from commercial closed-loop systems demonstrate the performance and acceptance of this technology outside of the trial setting [32]. Reassuringly, outcomes are similar to those reported in the pivotal studies in terms of efficacy (time spent in target glucose range and hypoglycaemia exposure) and usability (time spent in closed-loop mode). Comparisons between closed-loop systems with regards to efficacy and safety are limited by differences in the underlying characteristics of users.

Data from over 100,000 Medtronic 780G users across Europe, the Middle East and Africa showed that time spent in closed-loop mode was 90%, while time spent in target glucose range (3.9 to 10.0 mmol/l) was 72.3% with 2.0% of time spent below target range [33]. Real-world data show that optimal glucose management is associated with applying the lowest glucose target setting (5.5 mmol/l) and the shortest active insulin time setting (2 h) [34].

Data from over 20,000 Control-IQ users in the USA were analysed. The time spent in target glucose range was 71.5% with 1% time spent below target range. More aggressive correction factor settings, insulin-to-carbohydrate ratio settings and basal programs were all associated with higher time in range but also, to a lesser degree, to higher time below target [35]. Real-world usability has been reported in a smaller dataset (>9000 users) where time spent in closed-loop mode was 94% [36].

Adult Diabeloop users (>3500) in Germany spent 95% of time in closed-loop mode. The percentage time in target glucose range was 72.1% and time spent below 3.9 mmol/l was 0.9% [37].

Data from 1800 CamAPS FX users from 15 countries across a wide range of age groups showed that users spent 95% of time in closed-loop mode. Time in target glucose range was 72.6% and increased by age from 66.9% for users ≤6 years old to 81.8% for users ≥65 years. Time spent in hypoglycaemia (<3.9 mmol/l) was 2.3% [38].

A real-world dataset from nearly 70,000 Omnipod 5 users (aged ≥2 years) in the USA showed that users spent 94% of time in closed-loop mode, while time in target glucose range was 64.2% with 1.0% of time spent in hypoglycaemia (<3.9 mmol/l) [39].

A real-world dataset of 558 adults and children using open-source systems showed that the time spent in closed-loop mode was 83%. Time in target glucose range was 73% and time with glucose <3.9 mmol/l was 2.8% [40].

An NHS England pilot study evaluating real-world implementation of hybrid closed-loop technology included 251 children and 570 adults with type 1 diabetes [41, 42]. Of 226 children and young people with complete data at 6 months, HbA_1c_ was reduced by 0.7 percentage points and time in target glucose range (3.9 to 10.0 mmol/l) increased from 49% to 63%. There was also a significant reduction in time in hypoglycaemia. Of 520 adult users continuing hybrid closed-loop at 6 months, HbA_1c_ reduced by 1.7 percentage points, time in target glucose range increased from 34% to 62% and there was a significant reduction in time in hypoglycaemia.

## Challenging applications for closed-loop systems

While the clinical benefits of closed-loop systems appear to extend to all user groups, there are some populations where the need for glucose-responsive insulin delivery is particularly important. Data obtained from secondary analysis of closed-loop studies highlight specific populations where the day-to-day variability of insulin requirements are greatest, and therefore self-adjustment of insulin doses in response to glucose trends may be the most challenging.

### Adults with type 2 diabetes

**Fig. 2:**
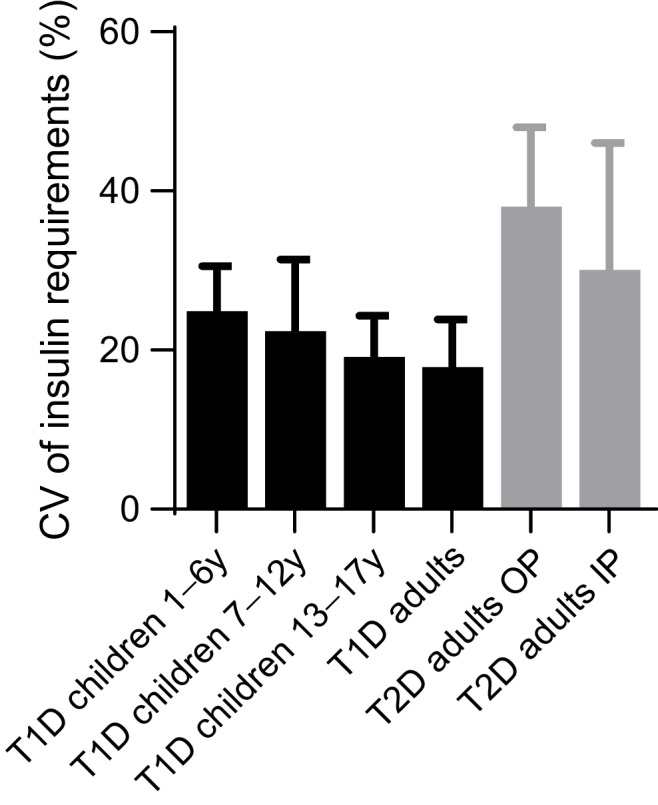
Variability of day-to-day insulin requirements as determined by closed-loop insulin delivery across different populations with diabetes. Data presented are mean with SD. Samples size: type 1 diabetes age 1–6 years *n*=20, 7–12 years *n*=21, 13–17 years *n*=18, adults *n*=58; type 2 diabetes outpatients *n*=25, inpatients *n*=67 (data from references [43, 44, 65]). IP, inpatients; OP, outpatients; T1D, type 1 diabetes, T2D, type 2 diabetes; y, years. This figure is available as part of a downloadable slideset

The population with the highest variability of day-to-day insulin requirements is adults with type 2 diabetes [43] (Fig. 2). This is perhaps surprising given the presence of residual endogenous insulin secretion, but perhaps explains the challenge that many people with type 2 diabetes and healthcare providers face in reaching glycaemic targets with standard insulin therapy. Inpatients with type 2 diabetes have lower variability of day-to-day insulin requirements than outpatients, despite the effect of the stress of acute illness, medications which exert effects on blood glucose levels and periods of fasting for inpatient investigations or procedures. This may reflect more sedentary behaviour and reduced oral intake during a hospital admission.

### Very young children with type 1 diabetes

The greatest variability in day-to-day insulin requirements in those with type 1 diabetes is in very young children [44] (Fig. 2). Additional challenges of inconsistent meal consumption, unannounced activity and inability to articulate symptoms of hypoglycaemia make this population arguably the most important to benefit from closed-loop systems. The impact on caregivers and other family members of very young children with type 1 diabetes is also profound [21].

### Pregnant women with diabetes

Attaining recommended tight glucose control during pregnancy is particularly difficult due to marked gestational variations in insulin sensitivity and altered eating patterns [45]. For pregnant women with type 1 diabetes, improved maternal glucose management is associated with reduced risk of large for gestational age infants, neonatal hypoglycaemia and neonatal intensive care unit admissions [46]. Recent evidence shows that the CamAPS FX hybrid closed-loop, which, unlike other commercially available systems, allows users to set glucose targets at the recommended pregnancy targets, improves time spent in the tight target glucose range (3.5 to 7.8 mmol/l) during pregnancy for women with type 1 diabetes, compared with standard insulin therapy, without increasing time spent in hypoglycaemia (<3.5 mmol/l) [47]. It remains to be seen if the same benefits are observed for pregnant women with type 2 diabetes or those with gestational diabetes requiring insulin.

### People with diabetes and impaired awareness of hypoglycaemia

Closed-loop systems may benefit individuals with long-standing diabetes where frequent exposure to hypoglycaemia impairs counterregulatory responses resulting in hypoglycaemia unawareness. In adults with type 1 diabetes at high risk for hypoglycaemia (Clarke score >3 and/or a history of severe hypoglycaemia during the previous 6 months), a hybrid closed-loop system significantly reduced time spent in hypoglycaemia (<3.9 mmol/l) compared with baseline, while improving time in target glucose range (3.9 to 10.0 mmol/l) [48]. Despite the reduction in time spent in hypoglycaemia, there was no improvement in self-reported hypoglycaemia awareness. Conversely, a small non-randomised study in ten adults with long-standing type 1 diabetes showed that use of a hybrid closed-loop system for 18 months reduced the frequency of severe hypoglycaemia events and improved hypoglycaemia awareness [49]. This was associated with increased adrenaline (epinephrine) secretion and improved autonomic symptoms in hypoglycaemic clamp studies.

### Frail people with diabetes and cognitive impairment

New challenges are arising in the optimal management of frail people, older adults and those with cognitive impairment who require assistance with their diabetes management. Closed-loop systems have not yet been robustly evaluated in this user group. Potential benefits of closed-loop systems in this population include operation using an off-body handset, availability of alerts for dangerous glycaemic excursions and the capability to review data remotely. Training of caregivers to use the closed-loop system is likely to be challenging, and acceptance of wearing the devices may be an issue for some people.

## Challenges to implementation of closed-loop technology

It is clear that closed-loop technology has brought positive life-changing experiences for many users. The greatest challenge at present is to ensure that these benefits can be extended widely to people living with diabetes, and in particular, more underserved populations: people from lower-middle or low-income countries, those from lower socioeconomic groups or ethnic minority groups and those with poorer health literacy or less access to health information.

Country-specific reimbursement policies are a key factor determining access to closed-loop systems. In England and Wales, recent National Institute for Health and Care Excellence (NICE) guidance mandates that hybrid closed-loop technology is recommended as an option for all children and young people with type 1 diabetes and for adults with type 1 diabetes and sub-optimal glucose management, or with disabling hypoglycaemia, despite best possible alternative management [50]. Reimbursement across European countries varies considerably from 100% reimbursement to very limited reimbursement, with or without a fixed out-of-pocket amount paid by the person with diabetes [51], and in the USA reimbursement is determined by insurance coverage or ability to meet costs.

Training received by users and the ongoing clinical support is an integral part of the closed-loop ecosystem (Fig. 1). To ensure widespread access to this technology, healthcare professionals need to be familiar with the rapidly expanding commercially available closed-loop systems. In addition, appropriate education material needs to be developed that is high quality, efficient and accessible to enable effective implementation. To support equitable roll-out the closed-loop training resources need to be available in multiple languages. While some training and support can be taken on by the closed-loop companies, some will inevitably fall on already overburdened healthcare providers. Healthcare professional workload, as well as bias as to who might use the closed-loop system most effectively and derive most benefit from it, are important contributors to inequalities in access to this technology for people with diabetes [52]. Standardised clinical outcome reporting on a common ‘diabetes data platform’ would facilitate users and healthcare professionals to get the most out of the technology, but is challenging with data protection legislation.

Regulatory hurdles can be onerous for the approval of new closed-loop devices (class III devices under EU Medical Device Regulation), particularly those with the capacity for interoperability (a unique regulatory classification devised by the US Food and Drug Administration [FDA]: alternate controller-enabled insulin pumps, integrated continuous glucose monitoring [CGM] systems and interoperable automated glycaemic controller devices). The lack of choice of interoperable devices at present may negatively impact the user experience. Other barriers to use of this technology include the physical burdens associated with wearing the devices and psychosocial burdens associated with alarm burden and device reliability issues. Usability, reflected by a sustained high time spent in closed-loop mode (>95%), is critical to realising the clinical benefits of closed-loop systems [53].

## What does the future of automated insulin delivery look like?

Closed-loop technology is fast becoming the standard of care for people with type 1 diabetes but work needs to be undertaken to ensure that barriers to access to closed-loop systems do not widen the current gap in health outcomes in underserved communities. The technology has the potential to bridge this gap if access is equitable and conscious and unconscious bias can be avoided.

Reducing device burden with smaller on-body devices (insulin pumps and sensors), longer duration wear time and improved reliability would increase the appeal of closed-loop systems. Expansion of interoperable closed-loop components, which offer wider user choice and customisation compared with integrated closed-loop systems, may also positively impact user experience.

Managing glucose excursions associated with meals and exercise remains challenging with current commercial hybrid closed-loop systems and future research needs to address these limitations. While hybrid closed-loop systems are the mainstay of automated insulin delivery, there is a growing role for artificial intelligence-generated insights and advice to support users in attaining optimal glucose management. This may be obviated if a safe and effective fully automated insulin delivery solution becomes commercially available. Potential solutions include the application of ultra-rapid-acting insulins with faster on and faster off profiles in closed-loop systems, dual hormone closed-loop systems, or adjuncts alongside closed-loop systems. Ultra-rapid insulins have the potential to improve closed-loop performance [54–57]; however, while the use of ultra-rapid Lispro in a fully closed-loop system reduced burden, this was at the expense of optimal glucose management [58]. Dual hormone closed-loop systems using insulin and pramlintide or insulin and glucagon have been evaluated in clinical trials but add complexity and cost, and are associated with gastrointestinal side effects [59, 60]. Adjuncts targeting postprandial glucose management, such as glucagon-like peptide-1 (GLP-1) receptor agonists and sodium–glucose cotransporter 2 (SGLT-2) inhibitors may allow for a fully closed-loop system, with small studies showing feasibility; however there are concerns around an increased risk of diabetic ketoacidosis [61, 62]. Given the immediate and marked improvement in glucose management observed after initiation of closed-loop technology, longer-term follow-up of users is required to investigate the potential development of diabetic retinopathy.

Innovative approaches to improving performance of closed-loop systems include incorporation of signals from other wearables such as a smartwatch app that detects eating behaviour [63] or a fitness sensor for detecting exercise/activity [64]. The potential clinical benefits of integrating these devices remains to be determined in larger and longer trials. Safety enhancements of closed-loop systems may include integration of a combined continuous glucose–ketone monitor or an infusion set failure detection system. Additional monitoring systems need to be accurate and reliable before incorporation into closed-loop systems.

In conclusion, closed-loop systems are enabling more people with type 1 diabetes to reach recommended glucose targets, but further improvements in performance and safety are needed. Reflecting the success of closed-loop technology for people with type 1 diabetes, there are emerging indications where automated insulin delivery may also benefit people living with other types of diabetes.

## Supplementary Information

Below is the link to the electronic supplementary material.

Supplementary file1 (PPTX 266 KB)

## Abbreviations

CFRD: Cystic fibrosis related diabetes
CGM: Continuous glucose monitoring
FDA: US Food and Drug Administration
