# Supplementary material for: The role of automated insulin delivery technology in diabetes
Source: Diabetologia. 2024 May 13;67(10):2034–44. doi: 10.1007/s00125-024-06165-w (PMC11457686; doi:10.1007/s00125-024-06165-w)
Supplement: Supplementary file 1 — Supplementary file1 (PPTX 266 KB) [file 125_2024_6165_MOESM1_ESM.pptx]

## Slide 1
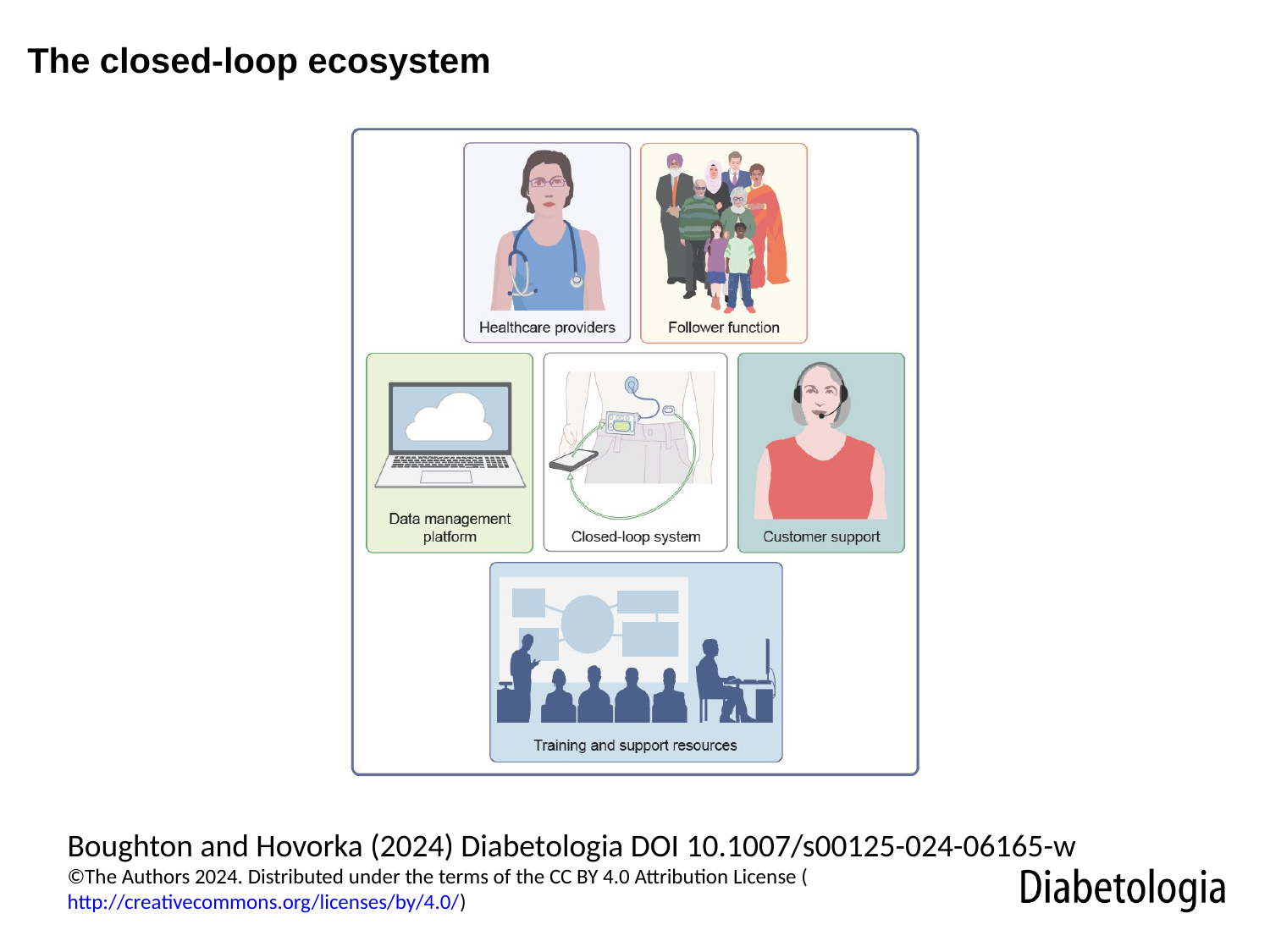

The closed-loop ecosystem
Boughton and Hovorka (2024) Diabetologia DOI 10.1007/s00125-024-06165-w
©The Authors 2024. Distributed under the terms of the CC BY 4.0 Attribution License (http://creativecommons.org/licenses/by/4.0/)

## Slide 2
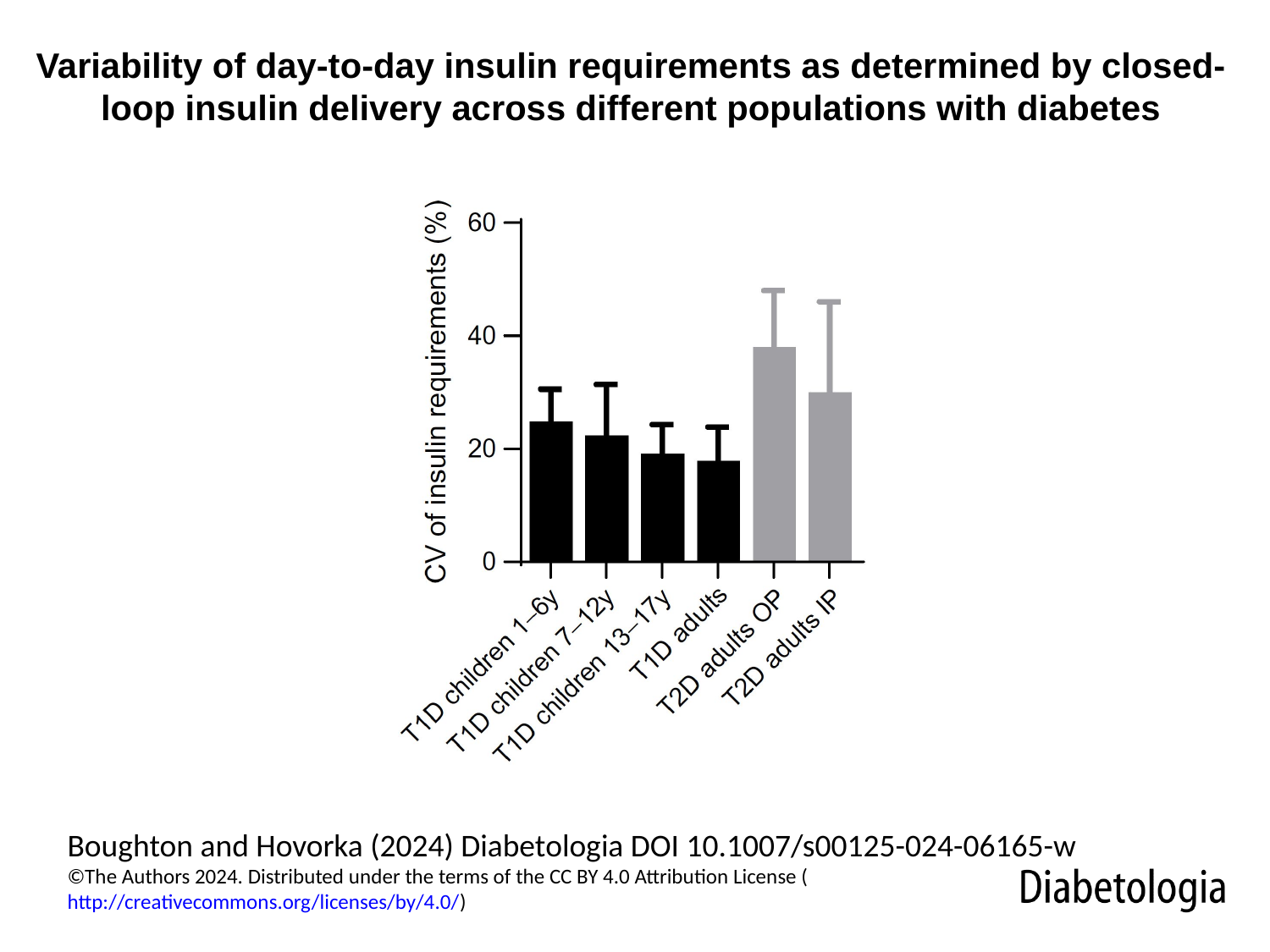

Variability of day-to-day insulin requirements as determined by closed-loop insulin delivery across different populations with diabetes
Boughton and Hovorka (2024) Diabetologia DOI 10.1007/s00125-024-06165-w
©The Authors 2024. Distributed under the terms of the CC BY 4.0 Attribution License (http://creativecommons.org/licenses/by/4.0/)
